# Barriers and facilitators affecting implementation of the Canadian clinical practice guidelines for the diagnosis of acute aortic syndrome

**DOI:** 10.1186/s43058-021-00160-7

**Published:** 2021-06-04

**Authors:** Caitlin Dmitriew, Robert Ohle

**Affiliations:** 1 Department of Undergraduate Medicine, Northern Ontario School of Medicine, Sudbury, Ontario Canada; 2 The Department of Emergency Medicine, Health Science North Research Institute, Northern Ontario School of Medicine, 41 Ramsey Lake Rd, Sudbury, ON P3E 5 J1 Canada

**Keywords:** Aortic dissection, Theoretical domains framework, Emergency medicine

## Abstract

**Background:**

Acute aortic syndrome (AAS) is an uncommon, life-threatening emergency that is frequently misdiagnosed. The 2020 Canadian clinical practice guidelines for the diagnosis of AAS incorporate all available evidence into four key recommendations. In order to facilitate the implementation of these recommendations, a clinical decision aid was created. The objective of this study was to identify barriers and facilitators among physicians prior to implementation of the guideline recommendations in a multicentre step wedge cluster randomized control trial.

**Methods:**

We conducted semi-structured interviews with nine emergency room physicians working at five sites distributed between urban academic and rural settings. We used purposive sampling, contacting physicians until data saturation was reached. Interview questions were designed to understand potential barriers and facilitators to guideline recommendation uptake and use. Responses were analysed according to the Theoretical Domains Framework, and overarching themes describing these barriers and facilitators were identified.

**Results:**

Two themes and six subthemes encompassing 13 theoretical domains were identified. These included clinical decision-making support, awareness of the evidence, social factors, expected consequences, ability of physicians to acquire the necessary data and ease of use. A majority of interviewees anticipated that the guideline recommendations would support clinical decision making and more effectively risk-stratify patients. Other facilitators included endorsement of the guidelines by professional organizations and peers. Barriers to implementation include the fact that laboratory testing and knowledge of the rationale for its use in the investigation of AAS were not widespread. The complexity of the clinical decision aid and concerns about test specificity were also identified as potential barriers to use.

**Conclusion:**

Physicians were amenable to using the AAS guideline recommendations to support clinical decision-making and to reduce resource use. A structured intervention should be developed to address the identified barriers and leverage the facilitators in order to ensure successful implementation. Our findings may have implications for the implementation of other guidelines used in emergency departments.

**Supplementary Information:**

The online version contains supplementary material available at 10.1186/s43058-021-00160-7.

Contributions to the literature

- Emergency physicians were interviewed to prospectively identify barriers and facilitators to implementation of the Canadian clinical practice guidelines to facilitate the diagnosis of acute aortic syndrome (AAS).
- Effective implementation of the recommendations may promote better outcomes and reduce unnecessary testing among patients being investigated for AAS.
- Findings can be extended to inform tailored strategies for the implementation of evidence-based guidelines used in emergency departments.

## Background

Acute aortic syndrome (AAS) refers to a group of life-threatening aortic pathologies including aortic dissection, intramural hematoma and penetrating atherosclerotic ulcer. This uncommon but potentially lethal condition is challenging to diagnose [1]. The low incidence of AAS, varied presenting symptoms, and lack of a standard diagnostic pathway has led to a misdiagnosis rate as high as 38% [2–4].

In order to address these challenges, Ohle et al. developed clinical practice guidelines for the diagnosis of AAS [5]. The guidelines incorporate all available evidence for the historical risk factors, patient symptoms, and physical exam findings associated with AAS into a clinical decision aid for risk stratification (see Additional file 1). Patients found to be at low risk require no further investigation for AAS, while those having a moderate pre-test probability can be further risk-stratified using a biochemical assay, the D-dimer test [6]. It is recommended that patients determined to be at high risk (corresponding to a pre-test probability of AAS greater than 5%) receive urgent imaging. The purpose of the guideline recommendations is to support clinical decision making in cases of suspected AAS, thereby minimizing diagnostic delays, misdiagnoses, and unnecessary advanced imaging.

Clinical guidelines are developed with the intention of standardizing clinicians’ approach to the diagnosis and treatment of disease, thereby improving patient outcomes and reducing resource overuse. However, they are only useful if incorporated into practice. The success of guideline implementation can be improved if the intervention addresses barriers and promotes behaviours or attitudes that will facilitate uptake. Interviewing end-users regarding the barriers and facilitators to guideline implementation is increasingly recognized as a crucial part of implementation planning and has been used to inform and evaluate strategies for maximizing the uptake of guidelines and clinical decision aids [7, 8]. The Theoretical Domains Framework (TDF) is a validated, integrative framework based on behavioural change theory that can be used to identify barriers and facilitators to behavioural change across 14 domains covering 84 theoretical constructs [9–11]. Data generated using this approach can be used to inform the design of implementation strategies that maximize the uptake of and adherence to both new and existing guidelines in the emergency department and beyond [8, 12–15].

In this paper, we report on the barriers and facilitators to the implementation of the new AAS clinical practice guidelines perceived by emergency physicians. Using the theoretical domains framework to guide our analysis of physician responses allows the barriers to be linked to the specific interventions most likely to facilitate guideline uptake and use [16, 17]. Adherence to clinical practice guidelines in this setting varies widely, but tailored interventions that address prospectively identified barriers may improve rates of adherence in a coming multicentre study [18–20].

## Methods

This study is part of a larger 10 site step wedge cluster randomized control trial for the implementation of the Canadian clinical practice guidelines for the diagnosis of AAS. A mixed deductive and inductive approach was taken to the analysis [21]. The initial data analysis involved a deductive content analysis in which utterances were coded into TDF domains. A subsequent inductive thematic analysis identified overarching themes that emerged among throughout the process of coding statements into TDF domains [22].

### Participants and setting

Participants were a purposive sample of practicing emergency room physicians from three academic centers and two rural emergency departments in Ontario (Table 1). These sites were a representative sample of the 10 planned implementation sites. Participants were recruited from each site by emailing emergency physicians at each site and inviting them to participate in a telephone interview. Based on prior studies, a predicted sample size of 8–12 participants was estimated, and interviews continued until no new themes had emerged for two consecutive interviews [7, 13, 14].

**Table 1:** Demographic information of ER physicians interviewed

	N
Sex	
Male	7
Female	2
Practice setting	
Full time urban academic	6
Part time urban and rural	3
Years in practice	
< 5	4
5–9	2
10–14	0
> 15	3
Mean # ER shifts per week	
< 3	2
3–4	5
> 4	2

### Interview procedure

Interviews were conducted by CD, a research assistant with no personal relationship to the participants, using a semi-structured approach (Additional file 2). Interviews took place between July and October 2019 and were audio recorded, transcribed verbatim and anonymized. The interview began with a detailed review of the decision aid and was followed by a series of open-ended questions designed to elicit the thoughts, beliefs and opinions regarding barriers and facilitators to implementing the guideline recommendations.

### Data analysis

The authors had not previously conducted studies applying the TDF and followed the guidance of Atkins et al. [10]. The first interview was coded collaboratively by the authors in order to establish a shared understanding of TDF domains and their definitions and to develop the initial coding guideline [9, 10]. Throughout the coding process, coding criteria were continuously reviewed and refined as described in the final coding guideline (Additional file 3). Subsequent interviews were coded independently in order to support trustworthiness of coding. When discrepancies in coding arose, they were resolved through discussion.

Coded utterances were then grouped into belief statements that represented similar responses across interviewees. Belief statements were generated by one reviewer (CD) and verified by another (RO), and supporting verbatim quotes are provided. Frequencies for each belief statement were compiled with each one being counted only once per interview. Thematic analysis of the belief statements generated from the TDF analysis was conducted according to the process outlined by Braun and Clarke [22].

## Results

Nine semi-structured interviews were conducted with practicing ER physicians (Table 1).

One hundred twenty-two utterances were coded into the 14 TDF domains, thirteen of which were identified as potentially influencing guideline recommendation uptake and accurate use. No utterances were coded into the domain Intention, as all physicians expressed some degree of interest in adopting the guideline recommendations. A variety of rationales were given for the stated intention to adopt or delay adopting the aid, and these were coded to the domain that best captured the specific reasoning. Belief statements generated from these utterances described barriers and facilitators to implementation (Table 2) and accurate application (Table 3) of the guideline recommendations. Within this data, two overarching themes encompassing six sub-themes were identified.

**Table 2:** Domains describing barriers and facilitators to decision aid implementation

TDF domain	Specific belief	Barrier or facilitator	Representative quotes (interview ID)	N
Knowledge	I need to see the evidence supporting the guideline before adopting it into practice	B	“it’s not clear to me that a zero on this score is associated with less than 5% chance of acute aortic syndrome. And then the same applies to each subsequent risk tier.” (5) “I think a validation of study of the decision aid would have to be done to convince people to use it, as opposed to using it off the bat with no validation study. But if the validation study was showing its utility then I think I would be amenable to using it.” (7)	6
	I am not convinced of the utility of D-dimer in screening for AAS	B	“Do I d-dimer all of them [moderate risk]? I can't say that I do. I probably do other tests that would decrease my suspicion before doing a d-dimer.” (4)	5
	The guidelines align with my understanding of the evidence for AAS risk factors	F	“I can't think of any additional factors that would prompt me to investigate for dissection other than what we already listed…it’s easy because it already fits with my mental model of aortic dissection.” (1)	4
	The clinical decision aid must be validated before I would use it	B	“You wonder about defensibility, as it is, especially … without, like, something like a randomized control trial to support it.” (6)	3
Behavioural regulation	Integrating new information into practice is cognitively challenging	B	“I would like to see evidence that stimulant use and hypertension are/are not important risk factors for AAS…sometimes it’s just hard to unlearn those things” (6)	1
Social influences	Guidelines help justify clinical decisions to colleagues	F	“Yeah, for sure [a guideline can] help make your case; speaking to other consultants and talking about ‘hey, have you heard of this new guideline that is actually supported by a bunch of other radiologists or vascular surgeons.’” (2)	2
	Decision aids support shared decision-making with patients	F	“I might say ‘we can do a d-dimer, and if it's negative [...] we've essentially ruled it out’. And it would be nice to be able to give them a number to say ‘if your d-dimer is negative, in a population of 100 people who are presenting similarly to you, only 0.5 percent of people are missed’, or whatever that number might be. I think for the patient to be engaged in that conversation, I need those numbers or I need the evidence to be able to have that conversation with them.” (5)	2
	More likely to use guidelines that have been endorsed by peers	F	“Getting the stuff published is usually successful, particularly studies that are published in decent journals, and decent meaning, respected journals.” (3) “Getting CAEP to endorse a set of rules is very influential in getting them incorporated, you know, when a group of emergency positions…a group that represents emergency physicians in Canada publishes the stuff, discusses it and says this is a standard of care—that's obviously very influential.” (3) “The fact that my colleagues aren’t also using it. I recently sort of polled like, a handful of people, and none of them are using d-dimer except for one, um, to help rule out aortic dissection in, like, low to medium risk patients. That is always a cause for concern–when you are doing something that is different than your colleagues.” (6)	5
Environmental context and resources	The guidelines will not be followed when CTs are readily available	B	“I don't think, honestly, they're going to be going for a decision aid, they’re going to say, get me the CT, because it's so easy for us.” (8)	1
	The guidelines will benefit small centers in particular because it may reduce the need for patient transport	F	“A decision rule that prevents people from needing complex investigations can be super useful particularly if you don't work in a major center.” (3)	3
Reinforcement	I am more likely to follow the guideline if it is shown to reduce resource use	F	“in the rural facility this is a really important opportunity to minimize transfer. So the effect on my practice, based on where the patient’s hanging out, whether we’re in [rural community] or something, I lose a nurse, I lose my physician assistant, if I send my patient out for CT scan.” (9)	2
Social/ professional role and identify	Clinical gestalt outperforms guidelines in clinical decision making	B	“I think a lot of times too with these decision aids, a lot of times it gets shown that clinical gestalt and experience is worth just as much as those, or perform just as well.” (8)	2
Beliefs about capabilities	Guidelines facilitate my decision making	F	“If I am uncertain and the patient is in a moderate risk category, then I would, I like the idea. I like the way this decision will makes you perhaps do a D dimer first as a screening test for CTA.” (3)	5
Beliefs about consequences	The clinical decision aid is likely to be very sensitive	F	“In all honesty I think your decision aid will make people happy, because it’s going to be hard to miss people, I think” (8)	2
	Guidelines will lead to an increase in imaging	B	“I think this may be one of these decision aids that will lead to increased testing, because it’s very easy to have one of the symptoms you’re talking about.” (8) “That heightened my concern about having a lot of positive d-dimers that then result in CT aortas being done… I would be worried that there would be a lot of false positives, which would lead to a lot more imaging being done.” (5)	7
Emotion	A clinical decision aid can help reduce anxiety around decision-making in AAS	F	“Cause right now I'm relying on my clinical gestalt. So it would be nice to have an evidence based tool to support my clinical decision making.” (5)	2
Optimism	Some cases of AAS will be missed even if the guidelines are followed	B	“Even if that same patient like my colleague had the other day came in again, I would probably be fooled again.” (1)	2
Goals	The guidelines will improve my ability to risk-stratify patients	F	“And I think the dimer offers me one more step to be able to risk-stratify these patients. Because otherwise, you either don’t do the test or you do the test-- like the CT--and it’s kind of like, well, we have another option now.” (9)	2

**Table 3:** Domains describing barriers and facilitators to accurate application of decision aid

TDF domain	Specific belief	Barrier or facilitator	Representative quotes (interview ID)	N
Skills	Physician vary in their ability to obtain the required physical exam findings required	B	“What I don't have is this clinical skill, and I don't think probably anybody in our department has the ability (or it would be very rare) to be able to measure aortic regurgitation or insufficiency.” (1)	4
Memory, attention and decision processes	The clinical decision aid is too long to memorize and requires an app	B	“It has to be published and … on MD calc. That would be useful. I don't think this is a tool you can memorize.” (4)	3
	The decision aid is difficult to use	B	“I get a little lost at first because the way you score each one is different” (1, 3) “When you pick up any kind of clinical decision rule we want to know if the patient’s meeting the inclusion criteria to begin with. CT head rule is a great example of that right? … it's a little misleading because it actually doesn't tell you who to apply the rule to. So not getting it confused with the components of the rule versus who to even apply the rule to.” (2)	4
	The decision aid is easy to use	F	“It's relatively easy to use, I mean the exclusions are very straightforward, that’s not hard to manage.” (9)	2
Environmental context and resources	Results of a D-dimer are fast and may accelerate care	F	“That's one of the advantages we have in our rural hospital, is that we have point of care testing, because we don't have lab 24/7.” (9)	2
	Waiting for a D-dimer may delay care	B	“One of the barriers, perhaps you could say, is the delay between investigating and getting a result.” (3)	2
	Required information may be unavailable	B	“A lot of this information we either don’t have, or we just have to assume.” (9)	2
Reinforcement	I will not order a D-dimer in patients for whom I suspect D-dimer will be positive for other reasons	B	“the patients I think are going to have a positive d-dimer for ten other reasons: I’m just going to go ahead and CT them… usually I just pull the trigger and make my day easier and order the scan upfront.” (1)	4
Beliefs about capabilities	Additional training/clarification is needed to correctly apply the clinical decision aid	B	“And I would hope that people are not saying, Oh, this person's blood pressure is 180 and they have some abdominal pain, therefore they go to CT. And I don't think that's the rule’s intent.” (3)	2
Beliefs about consequences	Subjectivity may lead to over-testing.	B	“is there potential that we will start over-investigating? Because a lot of patients will, for example, describe that their pain is severe, and so that automatically gives them, you know, one point…I think that severe pain requires a sort of a clinical judgment call.” (5) “One of my colleagues… CT scans a lot of people who I don’t necessarily think need it. So [they are] always going to say ‘aortic dissection is the most likely diagnosis’ whereas my cognitive bias is going to say ‘well, I think it's probably not’ … We're coming to a different conclusion from the same tool.” (1)	4

### I. Barriers and facilitators to implementation of the guideline recommendations

#### a. Decision-making support

*Relevant TDF domains: beliefs about capability, emotion, goals, optimism, social influences, social/professional role and identity*

Interviewees were largely receptive to the development of a clinical practice guideline for AAS. The reasons included a belief that the guideline recommendations were likely to reduce the number of missed cases of AAS, in part by forcing clinicians to consider the diagnosis more often, and the potential for D-dimer to safely reduce imaging rates by effectively risk-stratifying them prior to CT. Other perceived benefits of evidence-based guideline recommendations included reducing clinician anxiety, justifying clinical decisions (such as imaging orders) to colleagues, and aiding in shared decision making with patients.

The relative advantage of evidence-based guideline recommendations over clinical gestalt was controversial, with some physicians stating their conviction that guidelines are preferable, particularly for less experienced clinicians. Others expressed the belief that clinical gestalt is generally superior to guidelines, or that such tools are best used to supplement or guide clinical decision making rather than replacing it.

“I think a lot of times too with these decision aids… it gets shown that clinical gestalt and experience is worth just as much.” (Emergency Physician 8)

#### b) Awareness of the evidence

*Relevant TDF domains: knowledge; behavioural regulation*

A major theme was whether scoring criteria and follow-up investigation aligned or did not align with individual practice or understanding of the evidence. Of particular concern was the use of D-dimer as a screening tool in AAS. Several physicians were previously unaware of its potential application in AAS, and the majority of respondents expressed concern that the test was insufficiently specific given the variety of factors independent of AAS that may cause D-dimer to be elevated. Three respondents suggested that validation studies demonstrating the adequacy of D-dimer sensitivity and specificity in AAS were pre-requisites to adopting the guidelines into practice.

#### c) Social influences on the probability of adopting the guideline recommendations

*Relevant TDF domains: social influences*

Respondents were more likely to follow the guideline recommendations if it were published in a high-quality, peer-reviewed journal and endorsed by professional organizations representing medical specialties involved in the treatment of AAS. Uptake of the guidelines by departmental colleagues was also considered to be an important motivating factor.

#### d) Consequences of guideline use

*Relevant TDF domains: beliefs about consequences, optimism, reinforcement, environmental context and resources*

Many physicians felt that the guidelines were likely to increase the number of D-dimers ordered and lead to higher rates of CT scanning. Conversely, for physicians practicing in rural settings, the use of D-dimer screening was seen as a potential means for reducing patient transports to obtain imaging, which given the loss of accompanying ER staff, significantly strains rural emergency department capacity and workflow.

### II. Barriers and facilitators to accurate application of the guideline recommendations

#### a. Ability to acquire required necessary data

*Relevant TDF domains: skill; environmental context and resources*

Respondents expressed concern about their own ability or that of others to assess the physical exam findings used to risk-stratify patients, noting that variable proficiency in the use of point of care ultrasound among practitioners was likely to impact their ability to follow the guideline recommendations.

“What I don't have is this clinical skill, and I don't think probably anybody in our department has the ability…to measure aortic regurgitation or insufficiency.” (Emergency Physician 1)

Incomplete patient histories might similarly limit scoring accuracy; for example, patients may be unaware of the existence or nature of a pre-existing heart murmur or aortic valve disease.

Processing times for D-dimer varied among institutions, which influenced the probability of following the guideline recommendations accordingly. Some physicians were concerned that delays in D-dimer results were such that they might not wait to order a CT. On the other hand, at two rural facilities point of care D-dimer results were available immediately, such that clinicians viewed this step as a means of accelerating care for patients at risk of AAS.

#### b. Ease of use

*Relevant TDF domains: knowledge, memory, attention and decision processes; beliefs about capabilities, beliefs about consequences*

Physicians were divided in their response to the complexity of the guideline recommendations. Some reported that they found the clinical decision aid straightforward and easy to use. Other commented that the decision aid was too long to memorize, and several respondents suggested that a mobile app would be instrumental in promoting its use.

“I don't think this is a tool you can memorize.” (Emergency Physician 4)

Two physicians emphasized the importance of featuring inclusion criteria prominently at the top of the clinical decision aid, noting that this was a barrier to the correct use of existing decision aids. Finally, there was concern that subjectivity within the decision aid (specifically with regard to pain severity and probability of alternate diagnosis) was likely to increase scores and promote unnecessary testing.

## Discussion

In this study, we identified the barriers and facilitators likely to influence the implementation of the Canadian practice guideline for the diagnosis of AAS, with the goal of informing our intervention strategies for a multicentre implementation trial. Our findings also have broader implications for the design and uptake of guideline recommendations and decision aids in emergency departments in general.

Emergency physicians are required to make complex, cognitively challenging decisions regarding diagnostic assessment and management of patients in a crowded, time-constrained environment [23]. The development of clinical practice guidelines and decision aids is aimed at reducing practice variation and improving patient outcomes, while simultaneously minimizing unnecessary and potentially harmful testing.

Overall, physicians responded positively to the development of a guideline for the diagnosis of AAS because of the catastrophic nature of the condition and the diagnostic challenge it presents. The central barriers identified included factors that limited clinician capability (the capacity to adopt and use the guideline recommendations, including sufficient knowledge and skill) and motivation to adopt the guideline in light of concerns about its complexity and the specificity of D-dimer in the investigation of AAS.

### Implications for the implementation of guideline recommendations

The guideline recommendations were seen as having the potential to improve risk stratification of patients being investigated for AAS. Although designed specifically to inform clinical decision making by emergency physicians, this finding underscores the potential of the decision aid to support shared decision making with both colleagues and patients. Patients may thus derive additional benefits from the decision rule, including improved knowledge and a closer correspondence between their values and decisions [24].

Knowledge of the scientific rationale and pre-implementation skills training have repeatedly been cited as enablers to implementation of new guidelines and decision aids in the ED and other contexts [7, 12, 25]. Effective dissemination of the evidence underlying the decision aid is crucial, and respondents in the present study identified validation and publication of the guidelines in high quality journals as pre-requisites to uptake, as has previously been shown [17]. These challenges can be addressed by offering thorough pre-implementation training, continuing medical education credits and by providing a supporting appraisal of the evidence alongside the decision aid [26].

While physicians were confident about the likely sensitivity of the clinical decision aid, there was near universal concern that imaging could increase as a result of low specificity, impeding workflow and exposing patients to unnecessary radiation. Thus, in addition to validation studies investigating sensitivity and specificity of the clinical decision aid, the effect of the decision aid on imaging orders should also be assessed. The complexity of the decision aid was also perceived to be a barrier to use, particularly in a busy emergency department. Indeed, studies of other decision aids have shown that they are often used incorrectly in practice, being applied in patients that do not meet inclusion criteria used in validation studies or adding steps that were not included in the validated decision aid [7, 27, 28]. Evidence suggests that even relatively simple decision rules in common practice are difficult to remember [27]. The planned intervention must therefore incorporate a visually simple decision aid in which inclusion criteria are clearly presented at the outset. Environmental restructuring, such as facilitated access to copies of the clinical decision aid, an app, or integrating it into the electronic medical record, is also likely to support practice change.

### Strengths and limitations

The strengths of this study included the use of a theoretical framework to guide analysis of interview responses. Interviewees were selected from multiple institutions and represented diverse levels of experience and demographics in both urban and rural settings.

The limitations of the study included a relatively small sample size, though we ensured thematic saturation was reached by conducting two additional interviews after no new themes emerged. Interview questions were not designed to address specific theoretical domains, which may cause certain domains to be underrepresented in the data if they did not arise spontaneously. However, our approach increased the chances of identifying those barriers and facilitators that were the most salient to emergency physicians. Finally, given that the investigation was conducted prior to decision aid distribution and implementation, it is possible that unforeseen barriers will arise despite the attempt to address them *a priori*.

## Conclusions

Physicians were amenable to using the AAS decision aid due to its potential to support clinical decision-making and reduce resource utilization in a difficult to diagnose and lethal condition. Key barriers identified included the need for additional education and training prior to implementation of the guideline recommendations and concern about specificity of the decision aid criteria and D-dimer. Addressing these barriers pre-implementation through training, education and thoughtful design of the decision aid may improve integration of guideline recommendations into practice.

## Supplementary Information

**Additional file 1.** Acute aortic syndrome risk score.

**Additional file 2.** Interview script.

**Additional file 3.** Final coding manual.

**Additional file 4.** SRQR Checklist.

## Data Availability

All data generated or analysed during this study are included in this published article and its supplementary information files.

## References

[1] Evangelista A, Isselbacher EM, Bossone E, Gleason TG, Eusanio MD, Sechtem U (2018). Insights from the international registry of acute aortic dissection: a 20-year experience of collaborative clinical research. Circulation.

[2] Chua M, Ibrahim I, Neo X, Sorokin V, Shen L, Ooi SBS (2012). Acute aortic dissection in the ED: risk factors and predictors for missed diagnosis. Am J Emerg Med.

[3] Hansen MS, Nogareda GJ, Hutchison SJ (2007). Frequency of and inappropriate treatment of misdiagnosis of acute aortic dissection. Am J Cardiol.

[4] DeMartino RR, Sen I, Huang Y, Bower TC, Oderich GS, Pochettino A (2018). Population-based assessment of the incidence of aortic dissection, intramural hematoma, and penetrating ulcer, and its associated mortality from 1995 to 2015. Circ Cardiovasc Qual Outcomes.

[5] Ohle R, Yan JW, Yadav K, Cournoyer A, Savage DW, Jetty P (2020). Diagnosing acute aortic syndrome: a Canadian clinical practice guideline. CMAJ.

[6] Nazerian P, Mueller C, de Soeiro AM, Leidel BA, Salvadeo SAT, Giachino F (2018). Diagnostic accuracy of the aortic dissection detection risk score plus D-dimer for acute aortic syndromes: the ADvISED prospective multicenter study. Circulation.

[7] Curran JA, Brehaut J, Patey AM, Osmond M, Stiell I, Grimshaw JM (2013). Understanding the Canadian adult CT head rule trial: use of the theoretical domains framework for process evaluation. Implement Sci.

[8] Presseau J, Mutsaers B, Al-Jaishi AA, Squires J, McIntyre CW, Garg AX (2017). Barriers and facilitators to healthcare professional behaviour change in clinical trials using the theoretical domains framework: a case study of a trial of individualized temperature-reduced haemodialysis. Trials.

[9] Michie S, Johnston M, Abraham C, Lawton R, Parker D, Walker A (2005). Making psychological theory useful for implementing evidence based practice: a consensus approach. Qual Saf Health Care.

[10] Atkins L, Francis J, Islam R, O’Connor D, Patey A, Ivers N (2017). A guide to using the theoretical domains framework of behaviour change to investigate implementation problems. Implement Sci.

[11] Cane J, O’Connor D, Michie S (2012). Validation of the theoretical domains framework for use in behaviour change and implementation research. Implement Sci.

[12] Craig LE, McInnes E, Taylor N, Grimley R, Cadilhac DA, Considine J (2016). Identifying the barriers and enablers for a triage, treatment, and transfer clinical intervention to manage acute stroke patients in the emergency department: a systematic review using the theoretical domains framework (TDF). Implement Sci.

[13] Parks A, Eagles D, Ge Y, Stiell IG, Cheung WJ (2019). Barriers and enablers that influence guideline-based care of geriatric fall patients presenting to the emergency department. Emerg Med J.

[14] Crilly J, Greenslade JH, Berndt S, Hawkins T, Cullen L (2020). Facilitators and barriers for emergency department clinicians using a rapid chest pain assessment protocol: qualitative interview research. BMC Health Serv Res.

[15] Bunzli S, Nelson E, Scott A, French S, Choong P, Dowsey M (2017). Barriers and facilitators to orthopaedic surgeons’ uptake of decision aids for total knee arthroplasty: a qualitative study. BMJ Open.

[16] Michie S, van Stralen MM, West R (2011). The behaviour change wheel: a new method for characterising and designing behaviour change interventions. Implement Sci.

[17] Fischer F, Lange K, Klose K, Greiner W, Kraemer A (2016). Barriers and strategies in guideline implementation—a scoping review. Healthc Pap.

[18] Ebben RHA, Vloet LCM, Verhofstad MHJ, Meijer S, Mintjes-de Groot JAJ, van Achterberg T (2013). Adherence to guidelines and protocols in the prehospital and emergency care setting: a systematic review. Scand J Trauma Resusc Emerg Med.

[19] Baker R, Camosso-Stefinovic J, Gillies C, Shaw EJ, Cheater F, Flottorp S, et al. Tailored interventions to overcome identified barriers to change: effects on professional practice and health care outcomes. Cochrane Database Syst Rev. 2010:CD005470. 10.1002/14651858.CD005470.pub2 PMC4164371 20238340

[20] Baker R, Camosso‐Stefinovic J, Gillies C, Shaw EJ, Cheater F, Flottorp S, Robertson N, Wensing M, Fiander M, Eccles MP, Godycki‐Cwirko M, van Lieshout J, Jäger C. Tailored interventions to address determinants of practice. Cochrane Database Syst Rev. 2015;4(4):CD005470. 10.1002/14651858.CD005470.pub3. 10.1002/14651858.CD005470.pub3 PMC7271646 25923419

[21] McGowan LJ, Powell R, French DP (2020). How can use of the theoretical domains framework be optimized in qualitative research? A rapid systematic review. Br J Health Psychol.

[22] Braun V, Clarke V (2006). Using thematic analysis in psychology. Qual Res Psychol.

[23] Platts-Mills TF, Nagurney JM, Melnick ER (2020). Tolerance of uncertainty and the practice of emergency medicine. Ann Emerg Med.

[24] Stacey D, Légaré F, Lewis K, Barry MJ, Bennett CL, Eden KB (2017). Decision aids for people facing health treatment or screening decisions. Cochrane Database Syst Rev.

[25] Jabbour M, Newton AS, Johnson D, Curran JA (2018). Defining barriers and enablers for clinical pathway implementation in complex clinical settings. Implement Sci.

[26] Ebben RHA, Siqeca F, Madsen UR, Vloet LCM, van Achterberg T (2018). Effectiveness of implementation strategies for the improvement of guideline and protocol adherence in emergency care: a systematic review. BMJ Open.

[27] Brehaut JC, Stiell IG, Visentin L, Graham ID (2005). Clinical decision rules “in the real world”: how a widely disseminated rule is used in everyday practice. Acad Emerg Med.

[28] Stiell IG, Clement CM, Grimshaw JM, Brison RJ, Rowe BH, Lee JS (2010). A prospective cluster-randomized trial to implement the Canadian CT head rule in emergency departments. CMAJ.

