# Supplementary material for: Barriers and facilitators affecting implementation of the Canadian clinical practice guidelines for the diagnosis of acute aortic syndrome
Source: Implement Sci Commun. 2021 Jun 4;2:60. doi: 10.1186/s43058-021-00160-7 (PMC8178923; doi:10.1186/s43058-021-00160-7)
Supplement: Supplementary file 1 — Additional file 1. Acute aortic syndrome risk score. [file 43058_2021_160_MOESM1_ESM.docx]

**Acute aortic syndrome risk score**

Pre-test probability assessment and recommendations for investigation at a low risk (<0.5%), moderate risk (0.5-5%) and high-risk (>5%) pre test probability/prevalence. Inclusion - symptoms suggestive of acute aortic syndrome (chest, abdominal, back pain and/or perfusion deficit of central nervous system, cardiac, mesenteric or limbs); syncope, BP differential >20mmHg, Systolic >180mmHg).  Exclusion - <18 years, recent trauma, pregnant.

| **Risk factors**  –      Connective tissue disease  –      Aortic valve disease  –      Recent aortic manipulation  –      Family history of AAS  –      Aortic aneurysm | No risk factors | 0 |
| --- | --- | --- |
|  | Any non aneurysmal risk factors | 1 |
|  | Aortic aneurysm | 2 |
| **Pain**  –      Severe  –      Thunderclap  –      Tearing/ripping,  –      Migrating | No high risk pain | 0 |
|  | ≤2 high risk pain | 1 |
|  | >2 high risk pain | 2 |
| **Physical exam**  –      Pulse deficit  –      Neurological deficit  –      Aortic Insufficiency  –      Hypotension/ pericardial effusion | No high risk physical exam | 0 |
|  | - | - |
|  | Any high risk physical exam | 2 |
| **Alternative diagnosis**  *Acute coronary syndrome^1^, pulmonary embolism^2^ and stroke^3^ are the most common initial diagnosis for a missed case of AAS | Suspicion for an alternative diagnosis* | -1 |
|  | Unsure | 0 |
|  | AAS the most likely diagnosis | 1 |

**Low** – Score 0 – No further investigations

**Moderate** – Score 1 – D-dimer

**High** – Score  ≥2 – CT aorta

^1^ ACS + high risk pain/physical exam or risk factors for AAS– consider chest x-ray, point of care ultrasound +/- d-dimer

^2^ Pulmonary embolism + high risk pain/physical exam or risk factors for AAS hold anticoagulation until after CT

^3^ Stroke + high risk pain/physical exam or risk factors – CT/CTA including aortic arch
