# Supplementary material for: Barriers and facilitators affecting implementation of the Canadian clinical practice guidelines for the diagnosis of acute aortic syndrome
Source: Implement Sci Commun. 2021 Jun 4;2:60. doi: 10.1186/s43058-021-00160-7 (PMC8178923; doi:10.1186/s43058-021-00160-7)
Supplement: Supplementary file 2 — Additional file 2. Interview script. [file 43058_2021_160_MOESM2_ESM.docx]

**Interview script**

Thank you for agreeing to participate in a discussion today about managing patients with a suspicion for acute aortic syndrome. We are interested in your input to help implement a guideline for the management of these patients. Please feel free to give examples from your practice if it helps you answer a question.

The interview should take approximately 30 minutes and will be audio-recorded and transcribed. The transcription will be cleared of any identifying information. I will be the only person that will know what your answers were and will not share this information with anyone. The results will be published in aggregate form only, so responses cannot be traced back to you.

As we are asking for your thoughts and experiences, there are no right or wrong answers to the questions. If you prefer not to answer a question, or would like to withdraw from the study, you are free to do so at any time.

I am not a healthcare provider, so am not here to make any clinical judgments but may ask some questions that you find quite basic so that I can better understand the discussion.

Do you have any questions before we start?

Background questions

1. What is your job title?
2. How many years have you been in practice?
3. How would you describe your practice setting?
   1. Prompt: Academic hospital, Community hospital, Large/Small)
4. On average, how many days per week do you provide clinical care in your current emergency department?
5. How often do you see patients for whom you may have a clinical suspicion for acute aortic syndrome?
   1. Prompt: # per week that you think about it
6. My understanding is that when a patient presents to the ED with a suspicion for acute aortic syndrome, a physician will usually take a patient’s history, vitals, and physical exam. Would you do any testing in addition to this? What tests?
   1. Prompt: For example, bloodwork, ECG? Chest x-ray? D-dimer? And how frequently do you order these?
7. What factors do you consider when making a decision to investigate someone for acute aortic syndrome?
   1. Prompt: For example, specific features on history, clinical gestalt? Combination of factors? If so what?)

**[Review proposed decision aid]**

1. What is your initial reaction to this tool?

Now that we have reviewed the decision aid, we’d like to ask you about how the specific recommendations of this tool fit (or would fit) with your current practice investigating those with a suspicion for AAS. As we work through these questions, please let us know of any other issues that come to mind.

For our discussion today, we’d like to assume that we are only talking about patients - Patients >18 years, no trauma, no previous acute aortic syndrome.

LOW RISK PATIENTS.

The guidelines identify patients with a score 0 as LOW RISK, corresponding to a ≤ 0.5% of acute aortic syndrome within all patients in this group. For these patients, the decision aid recommends that these patients require no further testing for AAS. Can you think of a patient you had recently that would score low risk?

- Examples of patients who might score low risk:
  - Patient who presents with symptoms suggestive of AAS such as chest, abdominal or back pain and perfusion deficit, but has none of the risk factors, high risk pain symptoms or physical exam findings described in the clinical decision tool. Score = 0
  - Patient who presents with symptoms suggestive of AAS such as chest, abdominal or back pain and perfusion deficit, who describes pain as severe (+1 for 1-2 high risk pain symptoms) but who has no other risk factors or physical exam findings. You suspect an alternative diagnosis (-1). Score = 0

1. How does this recommendation compare to your current practice?
2. Keeping in mind the type of low-risk patients we are discussing, can you think of any barriers to applying the recommendations and not investigating these patients further
   1. Prompt: is there any clinical presentation or signs/symptoms that would prompt you to work the patient up further, despite the guideline’s recommendations?
3. What factors might aid you to incorporate these recommendations into your practice and investigate fewer patients in the low risk group?
4. Do you think patients should be involved in the decision to investigate when in a low risk group?
5. Are there any other issues you can think of that would affect your ability to adhere to the decision aid for low-risk patients?

MEDIUM RISK PATIENTS.

The guidelines identify patients with a score of 1 as MEDIUM RISK, corresponding to 0.5%-5% risk for AAS. For these patients, the decision aid recommends performing a D-dimer, if negative they are low risk and if positive they should undergo an ECG-gated CT aorta. Can you think of a patient you had recently that would score medium risk?

- Examples of patients who might score medium risk:
  - Patient who presents with symptoms suggestive of AAS such as chest, abdominal or back pain and perfusion deficit, and describes 1-2 high risk pain symptoms such as severe and tearing or ripping pain (+1). No other risk factors or physical exam findings. (Score = 1)
  - Patient who presents with symptoms suggestive of AAS such as chest, abdominal or back pain and perfusion deficit, with none of the high risk pain symptoms or physical exam findings, and who reports a history of aortic aneurysm (+2) but for whom you suspect an alternative diagnosis (-1). (Score = 1)

1. How do the above recommendations (D-dimer +/- ECG-gated CT aorta) compare to your current practice?
2. Keeping in mind the type of medium-risk patients we are discussing, what barriers might prevent you from following the guideline recommendations and performing a D-dimer?
   1. Prompt:
      1. Some physicians might worry about using D-dimer because it has a low specificity and might increase the number of CT that are performed
      2. Some physicians might worry about its sensitivity, it may miss a case of AAS
      3. Do you have access to blood tests 24hrs or CT 24hrs?
3. What factors might aid you to incorporate these recommendations into your practice and investigate with D-dimer in this medium risk group?
4. Do you think patients should be involved in the decision to order a D-dimer versus a CT first in a medium risk group?
5. Are there any other issues you can think of that would affect your ability to investigate medium-risk patients with D-dimer?
   1. Prompt:
      1. Are there any potential medium risk patients who you think should not have a d-dimer?
      2. Are there any patients at medium risk you think should have a CT directly?
      3. Are there any medium risk patients who you think does not need a d-dimer and requires no further investigation?

HIGH RISK PATIENTS.

The guidelines identify patients with a score of 2 and above as HIGH RISK, corresponding to >5% risk of AAS. For these patients, the decision aid recommends that patients should undergo an expedited ECG gated CT aorta. Can you think of a patient you had recently that would score high risk?

- Examples of patients who score high risk:
  - Patient who presents with symptoms suggestive of AAS such as chest, abdominal or back pain and perfusion deficit, describes 1-2 high risk pain symptoms (+ 1) and shows signs of pericardial effusion and hypotension on exam (+ 2). (Score = 3).
  - Patient who presents with symptoms suggestive of AAS such as chest, abdominal or back pain and perfusion deficit, with none of the high risk pain symptoms or physical exam findings, and who reports a history of aortic aneurysm (+ 2) and for whom you are unsure of the diagnosis (0). (Score = 2)

1. How do the above recommendations compare to your current practice?
2. Based on the risk criteria and the associated risk category on page 2, do you feel that high risk patients are appropriately categorized? Are there any patients that you might consider to be high risk but do not meet the criteria described here for high risk category patients.
   1. Prompt: For example, should a score of 4 be classified as high risk, or medium?
3. Keeping in mind the kinds of patients we are discussing, what barriers might prevent you from ordering a CT aorta?
   1. Prompt: e.g.
      1. Some physicians might have difficulty convincing radiology to perform a CT
      2. Some patients may not want to undergo a CT,
      3. Some physicians may not have access to CT/ call in a tech/ transport patient.
   2. (Prompt: Do you have access to ECG-gated CT?)
4. Can you think of any barriers that would affect your ability to investigate high risk patients with ECG-gated CT?
   1. Prompt: any clinical presentation or scenarios that would prevent you from ordering an ECG-gated CT?
5. What factors might aid you to incorporate these recommendations into your practice and perform ECG-gated CT in high risk patients?
6. Are there any other issues you can think of that would affect your ability to investigate high risk patients with ECG-gated CT?
7. Do you think high risk patients should be involved in the decision-making process to proceed with further investigation for AAS?

Final (general) questions

1. Can you think of any other issues related to using the decision aid to guide investigation decisions for patients with a suspicion for AAS?
2. Do you intend to incorporate the decision aid and the above recommendations in your clinical practice in the future? Is there a reason why or why not?
3. Is there anything else related to this topic you would like to talk about that we haven’t covered?
