# Supplementary material for: Barriers and facilitators affecting implementation of the Canadian clinical practice guidelines for the diagnosis of acute aortic syndrome
Source: Implement Sci Commun. 2021 Jun 4;2:60. doi: 10.1186/s43058-021-00160-7 (PMC8178923; doi:10.1186/s43058-021-00160-7)
Supplement: Supplementary file 3 — Additional file 3. Final coding manual. [file 43058_2021_160_MOESM3_ESM.docx]

**Final coding manual**

| TDF domain | Definition (Atkins et al. 2014) | Description |
| --- | --- | --- |
| Knowledge | An awareness of the existence of something   - Knowledge (including knowledge of condition/scientific rationale) - Procedural knowledge - Knowledge of task environment | - Awareness of the scientific evidence underlying the guidelines and decision aid - Awareness of the rationale for use |
| Skills | An ability or proficiency acquired through practice   - Skills - Skills development - Competence - Ability - Interpersonal skills - Practice - Skill assessment | - Physical skills required to use the decision aid - How easy is it for them to perform the steps required by the decision aid (e.g. components of the physical exam) |
| Memory, attention and decision processes | The ability to retain information, focus selectively on aspects of the environment and choose between two or more alternatives)   - Memory - Attention - Attention control - Decision making - Cognitive overload/tiredness | - How easy is it to follow the guidelines/decision aid - Under what conditions might physicians forget to use the decision aid or forget specific steps - What factors influence ability to follow the guidelines/decision aid or frequency of use |
| Behavioural regulation | Anything aimed at managing or changing objectively observed or measured actions   - - Self-monitoring   - Breaking habit   - Action planning | - Personal behaviours or characteristics that affect integration of the guidelines/decision aid into practice |
| Social influences | Norms; those interpersonal processes that can cause individuals to change their thoughts, feelings, or behaviours   - Social pressure - Social norms - Group conformity - Social comparisons - Group norms - Social support - Power - Intergroup conflict - Alienation - Group identity - Modelling | - Social influences (from peers, professional groups, patients and family) that affect use of the guidelines/decision aid - Influence of having role models/observing others |
| Environmental context and resources | Any circumstance of a person’s situation or environment that discourages or encourages the development of skills and abilities, independence, social competence and adaptive behaviour   - - Environmental stressors   - Resources/material resources   - Organisational culture/climate   - Salient events/critical incidents   - Person × environment interaction   - Barriers and facilitators | - Physical factors (including time and space) - Availability of resources that influence use of the decision aid |
| Reinforcement | Increasing the probability of a response by arranging a dependent relationship, or contingency, between the response and a given stimulus   - Rewards (proximal/distal, valued/not valued, probable/improbable) - Incentives - Punishment - Consequents - Reinforcement - Contingencies - Sanctions | - Incentives that promote or discourage a behaviour |
| Social/professional role and identity | Self-standards; a coherent set of behaviours and displayed personal qualities of an individual in a social or work setting   - Professional identity - Professional role - Social identity - Identity - Professional boundaries - Professional confidence - Group identity - Leadership - Organisational commitment | - Role of guidelines in determining a physician’s practices - Belief that guidelines are or are not compatible with professional standards |
| Beliefs about capability | Self-efficacy; acceptance of the truth, reality or validity about an ability, talent or facility that a person can put to constructive use)   - Self-confidence - Perceived competence - Self-efficacy - Perceived behavioural control - Beliefs - Self-esteem - Empowerment - Professional confidence | - Physician confidence in their ability to identify AAS - Confidence in ability to apply the decision aid - Factors that would support ability to use decision aid |
| Beliefs about consequences | Anticipated outcomes/attitude; acceptance of the truth, reality, or validity about outcomes of a behaviour in a given situation   - Beliefs - Outcome expectancies - Characteristics of outcome expectancies - Anticipated regret - Consequents | - Beliefs about possible outcomes (positive or negative) of guidelines use |
| Emotion | A complex reaction pattern, involving experiential, behavioural, and physiological elements, by which the individual attempts to deal with a personally significant matter or event   - Fear - Anxiety - Stress - Depression - Burnout - Positive/negative affect | - Emotional response provoked by AAS or by use of the decision tool - How emotional responses might facilitate or obstruct use of the guidelines/decision aid |
| Optimism | The confidence that things will happen for the best or that desired goals will be attained   - Optimism - Pessimism - Unrealistic optimism - Identity | - Optimism or pessimism regarding the likely outcomes of decision aid use |
| Goals | Mental representation of the desired outcome   - Goals (distal/proximal) - Goal priority - Goal/target setting - Goals (autonomous/controlled) - Action planning - Implementation intention | - What physicians hope to achieve in investigation of AAS - What they hope to achieve by using the guidelines/decision aid |
| Intentions | A conscious decision to perform a behaviour or a resolve to act in a certain way   - Stability of intentions - Stages of change model - Transtheoretical model and stages of change | - Intention to use the guidelines - Specific reasons for using/not using are coded accordingly (i.e. using it because it reduces anxiety is coded under “Emotion”). |
